# Social Transmission of Leadership Preference: Knowledge of Group Membership and Partisan Media Reporting Moderates Perceptions of Leadership Ability From Facial Cues to Competence and Dominance

**DOI:** 10.3389/fpsyg.2019.02996

**Published:** 2020-01-14

**Authors:** Christopher D. Watkins, Dengke Xiao, David I. Perrett

**Affiliations:** ^1^Division of Psychology, School of Applied Sciences, Abertay University, Dundee, United Kingdom; ^2^School of Psychology and Neuroscience, University of St Andrews, St Andrews, United Kingdom

**Keywords:** face perception, leadership, dominance, intelligence, priming

## Abstract

While first impressions of dominance and competence can influence leadership preference, social transmission of leadership preference has received little attention. The capacity to transmit, store and compute information has increased greatly over recent history, and the new media environment may encourage partisanship (i.e., “echo chambers”), misinformation and rumor spreading to support political and social causes and be conducive both to emotive writing and emotional contagion, which may shape voting behavior. In our pre-registered experiment, we examined whether implicit associations between facial cues to dominance and competence (intelligence) and leadership ability are strengthened by partisan media and knowledge that leaders support or oppose us on a socio-political issue of personal importance. Social information, in general, reduced well-established implicit associations between facial cues and leadership ability. However, as predicted, social knowledge of group membership reduced preferences for facial cues to high dominance and intelligence in out-group leaders. In the opposite-direction to our original prediction, this “in-group bias” was greater under *less* partisan versus partisan media, with partisan writing eliciting greater state anxiety across the sample. Partisanship also altered the salience of women’s facial appearance (i.e., cues to high dominance and intelligence) in out-group versus in-group leaders. Independent of the media environment, men and women displayed an in-group bias toward facial cues of dominance in same-sex leaders. Our findings reveal effects of minimal social information (facial appearance, group membership, media reporting) on leadership judgments, which may have implications for patterns of voting or socio-political behavior at the local or national level.

## Introduction

Social perceptions of faces influence a variety of important social outcomes (e.g., Fiske et al., 2007; Little et al., 2011; Vernon et al., 2014; Todorov et al., 2015) and are made rapidly (e.g., Todorov et al., 2005; Willis and Todorov, 2006; Engell et al., 2007; Carre et al., 2009; Olivola and Todorov, 2010), even, on some dimensions, when irrelevant to the task at hand (Ritchie et al., 2017). Complementing work on the role of social and physical dominance in leadership emergence and effectiveness (e.g., Rule and Ambady, 2008; Wong et al., 2011; Blaker et al., 2013; Hamstra, 2014; Pillemer et al., 2014; Rule and Tskhay, 2014;, see Watkins, 2018 for a recent review), first impressions of dominance and competence can guide leadership choice based on facial cues alone (Todorov et al., 2005; Ballew and Todorov, 2007; Little et al., 2007; Antonakis and Dalgas, 2009; Re et al., 2012, 2013; Olivola et al., 2014; Re and Perrett, 2014). Preferences for such traits in leaders may function to accrue fitness benefits for group members, if dominant, prestigious and/or intelligent leaders have the necessary leverage to represent or protect their group in exchanges with out-groups (see, e.g., Van Vugt et al., 2007; MacDonald et al., 2012; Spisak et al., 2012; for discussion), or to maintain cohesion, resolve conflict and/or enforce punishment within groups (see Watkins, 2018 for discussion). Collectively, social judgments of faces play a role in decisions as critical as our choice of political leader.

While the research reviewed above suggests that facial cues to dominance or competence can guide leadership choice, no work to our knowledge has examined the influence of social information in shaping these leadership judgments. This is an oversight given that appearance-driven biases may be stronger for certain types of voters (e.g., “undecided voters” see Todorov et al., 2015 for discussion) and money/effort invested in “marginal seats” can alter election outcomes (see Bond et al., 2012 for discussion). Research on social transmission of face preferences, and social attraction more generally, has examined the role of copying based on knowledge that people are desired by others (e.g., Jones et al., 2007; Place et al., 2010; Little et al., 2015; see Gouda-Vossos et al., 2018 for a recent review), copying the choices of those of good character (Chu, 2012) and attraction to others in light of knowledge of their intelligence (Gao et al., 2017; Watkins, 2017) and romantic relationship history (Quist et al., 2012). In addition, our experiences can influence social and/or romantic attraction to others, in light of previously cooperating or competing with them (Kniffin and Wilson, 2004; Faust et al., 2018) and when encountering people who resemble someone associated with an event that varied in its level of affect (Verosky and Todorov, 2010). Collectively, first impressions derived from facial cues are “offset” or strengthened by the experiences we have with those individuals.

In the domain of leadership and politics, new media provide one avenue to examine social transmission of leadership preference. A key transition in gene-culture coevolution (e.g., Gintis, 2011) is the world’s increased capacity to store, compute and transmit information (Hilbert and Lopez, 2011). With these advances, behavioral scientists have raised concerns about the extent to which misinformation and rumor are transmitted online via new media to support various social or political causes (reviewed in Lewandowsky et al., 2012). Indeed, some direct analyses of communication within online social networks supports the concept of an “echo chamber” (Del Vicario et al., 2016) where users expose themselves selectively to information that supports their pre-existing views. In general, motivated reasoning to support our personal, political and group interests is a well-established phenomenon (see Kunda, 1990; Mercier and Sperber, 2011; Haidt, 2012; for reviews), with meta-analyses suggesting a moderate preference for selecting information that supports versus challenges pre-existing beliefs, particularly when our confidence in a belief or attitude is low (Hart et al., 2009). Thus, new media may amplify such biases given that users can create their own content, send, and access view-consistent information quickly.

In the culture of new media, it is also worth noting that there is an affective component to acquiring information about the world around us, which may have contagious effects on behavior in social networks (e.g., Hill et al., 2010), including voting behavior (Bond et al., 2012). For example, emotions conveyed by social media users can alter the mood of their network members indirectly (Coviello et al., 2014) and encountering fewer positive or negative emotional posts in news feeds may alter network members’ posting behavior toward more negative or positive posts, respectively (Kramer et al., 2014). In politics, our own mood, task performance and evaluations of leader charisma appear to be strengthened by positive mood in leaders (Johnson, 2009). How leaders, debates and positions are described via media may therefore have “contagious” effects on leadership judgments, particularly if news sources are partisan and activate the “group-ish mind” (Haidt, 2012). Some early cross-cultural work suggests that market factors may be sufficient to shape the tone of news coverage, toward that which is more arousing (Vettehen et al., 2012). Moreover, sentiment analyses of news coverage suggest an historical trend toward coverage that is more negative in tone (Leetaru, 2011). This context/climate may therefore be important for social transmission of leadership preference if, for example, partisan sources strengthen biases toward a particular candidate/position and affective dimensions of the spoken or written message (high valence) activate concepts of cohesion and similarity (see Koch et al., 2016 for discussion). Collectively, both partisanship and affect may influence opinions on important social issues outside of our awareness, potentially qualifying the effects of appearance on leadership judgments.

Here, we examine whether partisanship, facilitated via new media, qualifies implicit associations between facial cues and leadership ability, focusing on the trait dimensions of dominance and competence (i.e., intelligence). Both of these traits derived from faces are important in leadership judgments (e.g., Todorov et al., 2005; Ballew and Todorov, 2007; Little et al., 2007; Antonakis and Dalgas, 2009; Re et al., 2012, 2013; Olivola et al., 2014; Re and Perrett, 2014). Thus, we predict that implicit associations between these traits and leadership ability will increase when judges have knowledge that individuals support their cause compared to when judges have knowledge that individuals oppose their cause on a socio-political issue of concern (i.e., knowledge of group membership, Hypothesis #1). Specifically, we predict that these judgment effects will be qualified by additional access to partisan information, such that changes in preference for facial cues to dominance/intelligence in leaders will be greater when participants are primed with partisan information compared to when they are primed with less partisan information (Hypothesis #2). Evidence for these predictions would suggest that there is a stronger bias to use minimal cues that denote good leadership (facial characteristics) for strategic advantage, when primed with cultural information that facilitates a “groupish” mind-set (Haidt, 2012). Finally, in order to test the proposal that undecided voters are more prone to bias in leadership perceptions based on minimal information (see Todorov et al., 2015) we examine whether a trait-level measure of decisiveness moderates these two predictions, such that any predicted changes are greater in less-decisive individuals than they are in relatively decisive individuals (Hypothesis #3).

## Materials and Methods

### Participants

Two hundred and seventeen participants (107 males, 108 females, 2 other/undisclosed gender, *M*_age_ = 26.45 years, *SD* = 5.22 years) took part in the online experiment hosted via prolific academic. This hosting platform generates reliable data (Peer et al., 2017) and the current experiment follows previous online experiments that have demonstrated effects of priming on social judgments of faces (e.g., Watkins and Jones, 2012, 2016). Our local Ethics Committees approved all procedures for testing and recruitment (Approval Code: PS14273), with methods and hypotheses pre-registered before data collection via the Open Science Framework^1^. Our target sample size of two hundred sixteen participants (with even gender balance) was based on 90% power to detect a medium effect (Lakens and Evhers, 2014) with two between-subjects conditions in our design (sex of participant and experimental priming condition). We used the platform’s screening tool to select British participants aged 18–35 years and reimbursed participants the equivalent of £5 per hour. After excluding participants who did not identify as male or female, adhere to the instructions during the priming phase, or complete all trials or items on the face judgment task or decisiveness questionnaire, data were analyzed from 210 participants (107 males, 103 females, *M*_age_ = 26.36 years, *SD* = 5.19 years).

### Face Stimuli

We used 48 Caucasian faces (24 males, 24 females), with prior ratings of dominance/intelligence made by an independent panel of judges (Karolinska Directed Emotional Faces; Lundqvist et al., 1998; standardized z score ratings from Oosterhof and Todorov, 2008). Images were selected from this database such that, for each sex and trait (i.e., 12 images per trait-sex combination), six faces images were of individuals rated relatively high in intelligence or dominance and six face images were of individuals rated relatively low in intelligence or dominance. High/low face image sets were created by selecting faces from above/below the mean, respectively, with unique identities used across the dominance and intelligence image sets. The difference between “high” and “low” face image sets reflects approximately one standard deviation in ratings of that trait (Range 1.01–1.08 *SD*s). In the post-priming phase of the experiment, half of the six images in the high and low dominance/intelligence image sets were allocated to represent individuals from the side a participant supports on a socio-political issue (labeled Group A), while the other half of these six images were allocated to represent individuals from the side the participant opposes on the same socio-political issue (see Procedure). The difference between Group A and Group B face images (three face images for each combination of trait, sex and group) reflect approximately one standard deviation (Range = 0.98–1.10 *SD*s) difference in ratings of that trait (dominance or intelligence).

### Procedure

The experiment consisted of four phases: A pre-priming leadership perception task, a priming phase, a post-priming leadership perception task and a questionnaire phase. First, participants were asked to rate faces for leadership ability based on their first impressions. Participants were asked on each trial to indicate how good a leader they think the person is on a sliding scale of 0 to 10.0, with the scale integers not visible to the participant and the slider starting at a random position. Trial order in this 48-trial task was randomized.

Next, participants completed one of two priming conditions (partisan, less partisan). Participants were instructed to imagine that they are spending some time online reading about a particular social or political issue that is important to them. They were informed that there are two positions in this debate, where Group A represents the side they would tend to support, on balance, and Group B represents the side they would tend to oppose, on balance. Participants were guided that if they consider themselves “open minded” on the issue, that it may help to think of a side they would pick if forced to choose a side in a debate. Participants considered a range of issues during this phase of the experiment (United Kingdom independence from EU = 24%, Other issues related to government/politicians = 18%, Abortion = 17%, Other social/moral issues = 17%, Climate change/environment = 14%, Equality = 10%).

**FIGURE 1:**
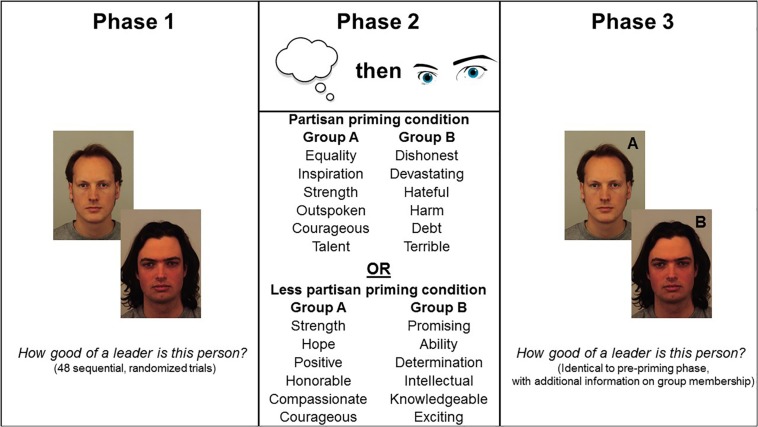
Experimental procedure, with stimuli used in experimental priming conditions.

Following these instructions, participants were informed that the following two lists of words have been used by the writer (i.e., a single writer) and are associated with the two sides in the debate (Figure 1). Participants viewed one of two pairs of word lists on the same page (Partisan condition: *N* = 53 males, 51 females. Less-partisan condition: *N* = 54 males, 52 females). The words constituting each pair of lists were extracted from a large publically available stimulus set rated on the three dimensions of affect (Warriner et al., 2013), where high scores (on a 1–9 scale) indicate happiness (valence), excitement (arousal) and feeling “in control” (dominance). From this list, we selected words to ensure that arousal was equivalent in both conditions when reading about Group A versus Group B, however, to manipulate partisanship, both valence and dominance differ significantly in favor of the side supported (Group A) versus the side opposed (Group B). In other words, both words lists in each condition are equally arousing (Partisan condition: Arousal *M*_GroupA_ = 5.21, *SD* = 0.37, Arousal *M*_GroupB_ = 5.47, *SD* = 0.57; *t*(10) = 0.94; *p* = 0.37. Less-partisan condition: Arousal *M*_GroupA_ = 5.18, *SD* = 0.35, Arousal *M*_GroupB_ = 5.20, *SD* = 0.93; *t*(10) = 0.04; *p* = 0.97) but, in the partisan condition only, it is more pleasant to read about the side supported versus opposed (Partisan condition: Valence *M*_GroupA_ = 6.75, *SD* = 0.68, Valence *M*_GroupB_ = 2.00, *SD* = 0.09; *t*(10) = 17.05; *p* < 0.001. Less-partisan condition: Valence *M*_GroupA_ = 7.58, *SD* = 0.47, Valence *M*_GroupB_ = 7.51, *SD* = 0.44; *p* = 0.82) and readers should feel a greater sense of control when reading about the side they support versus the side they oppose (Partisan condition: Dominance *M*_GroupA_ = 6.51, *SD* = 0.69, Dominance *M*_GroupB_ = 3.94, *SD* = 0.36; *t*(7.47) = 8.06; *p* < 0.001. Less partisan condition: Dominance *M*_GroupA_ = 7.05, *SD* = 0.35, Dominance *M*_GroupB_ = 6.82, *SD* = 0.38; *p* = 0.30).

After reading the word lists (i.e., Group A and Group B simultaneously on the screen), participants were asked how they would feel in that moment as they are reading the article, using an adapted version of the short-form state anxiety inventory (i.e., as a manipulation check; Marteau and Bekker, 1992). As expected, state anxiety was greater in the partisan condition (*M* = 0.14, *SD* = 1.27) than in the less partisan condition (*M* = 0.82, *SD* = 1.22, absolute *t*(206) = 3.94, *p* < 0.001, *r* = 0.26, low scores denote high anxiety).

Immediately following on from the priming phase of the experiment, participants were asked to rate faces for leadership ability. This phase was identical to the pre-priming leadership perception task except that half of the face images were of individuals from Group A and half of the face images were of individuals from Group B (labels “A” or “B” were used in black text in the right corner of each face image on each trial). Participants were told that some of the faces will be from Group A (the side you support) and some of the faces will be from Group B (the side you oppose). Participants were asked to confirm that they understand this via tick-box before proceeding to the face rating task. To ensure attention to the group labels during this phase, we informed participants that they might be asked questions about the faces after the task (which group they belong to). Finally, in the questionnaire phase of the experiment, participants were asked to complete a self-report measure of general decisiveness (Germeijs and De Boeck, 2002). This 22-item measure consists of items such as “I delay deciding” and “I don’t hesitate much when I have to make a decision”. Participants were informed that the questionnaire concerns decision making in general, in all kinds of situations, and were asked to indicate the extent to which they agree with each statement on a 0 (strongly disagree) to 6 (strongly agree) scale, with item order randomized and high scores indicating high trait decisiveness (*M* = 3.30, *SD* = 0.92, Range = 0.23–6.00). Following this, participants were debriefed and exited the task.

### Initial Processing of Data

Participants’ responses to both pre- and post-priming phases of the experiment were used to calculate their preference for facial cues to dominance/intelligence in leaders, separately when judging female faces and male faces and separately for faces used as members of Group A and members of Group B (i.e., eight scores for each of the two traits judged). Scores were calculated by taking the average preference, across trials, for high-dominance/intelligence leaders minus the average preference, across trials, for low dominance/intelligence leaders. Then, we calculated each participant’s change in preference for facial cues to dominance/intelligence by subtracting their pre-priming scores from their respective post-priming scores. Scores above/below zero indicate a respective increase or decrease in the tendency to associate facial cues to intelligence/dominance with leadership ability following priming with media information and social knowledge of the pictured individual’s group membership. Although we did not pre-register hypotheses for effects of the sex of participant in the experiment or sex of face rated in the task, these are included as factors in our models in order to examine whether our pre-registered predictions generalize across sex, or are specific to a given sex of face or participant. Such differences may be observed, for example, in light of sex-specific responses to faces in related domains (e.g., when judging allies; Watkins and Jones, 2016) and the evolutionary theory underpinning related work (i.e., male intrasexual competition, see, e.g., Watkins, 2018 for discussion).

## Results

**FIGURE 2:**
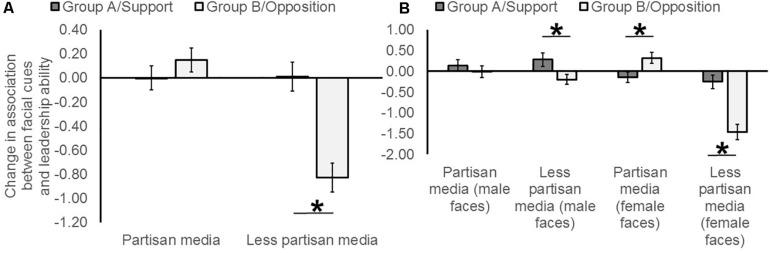
**(A)** The predicted effect of group (Hypothesis #1) was qualified by experimental priming condition (Hypothesis #2) in the opposite direction to that predicted (a relative in-group bias under less-partisan media, *r* = 0.31). **(B)** Partisanship moderated the salience of appearance cues in out-group versus in-group female leaders to a greater extent than it did for male leaders. The asterisks indicate significant effects of group.

An initial one-sample *t*-test against chance (i.e., zero) revealed that, in general, our priming manipulation (media information and knowledge of group membership) *reduced* implicit associations between facial cues and leadership ability (*M* = −0.18, *SEM* = 0.07, *t*(196) = −2.66; *p* = 0.008, *r* = 0.10). A mixed ANOVA, on the dependent variable *change in preference for facial cue in leaders*, with the within subjects factors *facial characteristic* (cue to high dominance, cue to high intelligence), *group* (support/“group A”, opposition/“group B”) and *sex of face* (male faces, female faces) and the between subjects factors *experimental priming condition* (partisan media information, less-partisan media information) and *sex of participant* (men, women) revealed a main effect of *group* [*F*(1,193) = 14.50; *p* < 0.001, np^2^ = 0.07] and a main effect of *experimental priming condition* [*F*(1,193) = 14.81; *p* < 0.001, np^2^ = 0.07], with these two factors interacting [*F*(1,193) = 30.24; *p* < 0.001, np^2^ = 0.14]. This interaction reflected a relative in-group bias toward facial cues to leadership ability (dominance and intelligence) when the media were less partisan (Change: *M*_Group A/Support_ = 0.01, *SEM* = 0.12, *M*_Group B/Opposition_ = −0.83, *SEM* = 0.12, absolute *t*(101) = 6.67; *p* < 0.001, *r* = 0.31) but not when they were partisan (Change: *M*_Group A/Support_ = −0.0004, *SEM* = 0.10, *M*_Group B/Opposition_ = 0.15, *SEM* = 0.10, absolute *t*(94) = 1.17; *p* = 0.24, Figure 2A). *Experimental priming condition* also interacted with *sex of participant* [*F*(1,193) = 5.03; *p* = 0.026, np^2^ = 0.03], which reflected women’s increased preference for facial cues to leadership ability when the media are partisan (Change: *M*_Partisan_ = 0.13, *SEM* = 0.11, *M*_Less partisan_ = −0.63, *SEM* = 0.13, absolute *t*(98) = 4.52; *p* < 0.001, *r* = 0.42), with no corresponding bias among men (Change: *M*_Partisan_ = 0.02, *SEM* = 0.11, *M*_Less partisan_ = −0.18, *SEM* = 0.15, absolute *t*(87.93) = 1.11; *p* = 0.27).

A main effect of *sex of face* was observed [*F*(1,193) = 18.57; *p* < 0.001, np^2^ = 0.09], which interacted with *experimental priming condition* [*F*(1,193) = 20.45; *p* < 0.001, np^2^ = 0.10]. These two factors were involved in a further three way interaction with *group* [*F*(1,193) = 14.42; *p* < 0.001, np^2^ = 0.07], which reflected an in-group bias in the use of facial cues to female leadership ability when the media were less partisan (Change: *M*_Group A/Support_ = −0.25, *SEM* = 0.16, *M*_Group B/Opposition_ = −1.46, *SEM* = 0.18, absolute *t*(101) = 6.94; *p* < 0.001, *r* = 0.33) and an out-group bias in the use of facial cues to female leadership ability when the media were partisan (Change: *M*_Group A/Support_ = −0.14, *SEM* = 0.13, *M*_Group B/Opposition_ = 0.32, *SEM* = 0.13, absolute *t*(94) = 3.00; *p* = 0.003, *r* = 0.15). For male faces, we observed an in-group bias in the use of facial cues to leadership ability when the media were less partisan (Change: *M*_Group A/Support_ = 0.28, *SEM* = 0.16, *M*_Group B/Opposition_ = −0.20, *SEM* = 0.12, absolute *t*(101) = 2.51; *p* = 0.014, *r* = 0.12) but not when they were partisan (Change: *M*_Group A/Support_ = 0.14, *SEM* = 0.14, *M*_Group B/Opposition_ = −0.01, *SEM* = 0.14, absolute *t*(94) = 0.80; *p* = 0.43). The three way interaction reported here demonstrates that the effect observed in female faces (in-group bias under less partisan media) was significantly greater than the corresponding effect observed in male faces (Figure 2B).

A three-way interaction was also observed between *facial characteristic*, *sex of face* and *group* [*F*(1,193) = 4.52; *p* = 0.035, np^2^ = 0.02], which was involved in a further four-way higher order interaction with *sex of participant* [*F*(1,193) = 5.99; *p* = 0.015, np^2^ = 0.03]. No other effects or interactions in the model were significant (all *F* < 3.68 all *p* > 0.057). Paired *t*-tests to interpret this four way interaction revealed that both men (Change: *M*_Group A/Support_ = 0.38, *SEM* = 0.16, *M*_Group B/Opposition_ = −0.15, *SEM* = 0.19, absolute *t*(96) = 2.16; *p* = 0.033, *r* = 0.11) and women (Change: *M*_Group A/Support_ = −0.21, *SEM* = 0.17, *M*_Group B/Opposition_ = −0.76, *SEM* = 0.20 absolute *t*(99) = 2.44; *p* = 0.017, *r* = 0.12) displayed an in-group bias toward facial cues to dominance in same-sex leaders but not opposite-sex leaders (both absolute *t* < 1.27, both *p* > 0.20). Although both men and women had a stronger in-group bias toward facial cues to intelligence in female leaders but not male leaders (both absolute *t* < 1.70, both *p* > 0.09), this bias toward female leaders was statistically significant among men (Change: *M*_Group A/Support_ = −0.06, *SEM* = 0.19, *M*_Group B/Opposition_ = −0.87, *SEM* = 0.22, absolute *t*(96) = 3.64; *p* < 0.001, *r* = 0.18) but not among women (Change: *M*_Group A/Support_ = −0.27, *SEM* = 0.24, *M*_Group B/Opposition_ = −0.77, *SEM* = 0.28, absolute *t*(99) = 1.97; *p* = 0.052, *r* = 0.10).

As our pre-registered Hypotheses (#1 and #2) were qualified by higher order interactions, we examined (Hypothesis #3) whether decisiveness was correlated with (i) the dependent variable for each cell within the predicted *group* x *experimental priming condition* interaction, and (ii) the general change in implicit associations between facial cues and leadership ability across the sample following priming (exploratory correlational test to follow up the initial one sample *t*-test). Correlational tests split by priming condition revealed no significant relationships between trait-level decisiveness and these dependent variables (all absolute *r* < 0.13, all *p* > 0.25).

## Discussion

Our experiment revealed that priming partisanship had a direct effect in enhancing self-reported state anxiety. Moreover, as participants were not aware that our rated image set varied on two trait dimensions, social knowledge of group membership and media information, regardless of partisanship, had a general effect in *reducing* implicit associations between perceived leadership ability and facial cues to dominance and intelligence. Our first prediction (Hypothesis #1) was supported, as social knowledge of group membership reduced preferences for facial cues to leadership ability (dominance and intelligence) in leaders who opposed the participant on their imagined socio-political debate (Group B), while preferences for “in-group” leaders (Group A) remained almost unchanged. In addition, our second prediction was supported (Hypothesis #2), albeit in the opposite direction to that originally predicted. Here, we observed a relative in-group bias toward facial cues to leadership ability when the media were *less partisan* but not when the media were partisan (Figure 2A). This suggests that appearance cues may afford an advantage for members of a socio-political group in light of perceptions derived from the media (i.e., where media portray two groups as similar on the valence and dominance dimensions of affect). Finally, our predictions were not moderated by trait-level decisiveness (Hypothesis #3), in contrast to recent discussion where low levels of this trait may explain stronger tendencies toward appearance-driven biases (Todorov et al., 2015).

Although not part of our pre-registered predictions, other interactions were observed in our model. First, independent of the group the pictured individuals belonged to, women had a stronger bias than men in preferring facial cues to leadership ability under partisan versus less partisan media. Second, the sex of the leader also explained priming-induced changes in ratings of faces. Here, we observed an in-group bias in the use of facial cues to leadership ability under less partisan media, which was stronger when judging female leaders than male leaders. Indeed, among female leaders, while we observed an in-group bias in the use of facial cues to leadership ability under less partisan media, we observed a relative *out-group* bias in the use of facial cues to leadership ability under partisan media. Thus, appearance cues in female leaders may be used to create a strategic advantage under less partisan media, while these same facial cues (dominance and intelligence) are salient in out-group females when partisan media portray this group negatively. Finally, complex interactions between the sex of the leader and rater were observed in response to specific facial cues. Here, while men and women displayed an in-group bias toward facial cues of dominance in same-sex leaders, men had a stronger bias than women toward intelligent-looking in-group females. These data suggest that perceived dominance may be an important cue in same-sex allies within socio-political groups, while men have a stronger bias than women toward competent-looking female leaders.

Our findings develop the literature on perceptions of leadership from facial cues related to dominance and competence (Todorov et al., 2005; Ballew and Todorov, 2007; Little et al., 2007; Antonakis and Dalgas, 2009; Re et al., 2012, 2013; Olivola et al., 2014; Re and Perrett, 2014), by providing direct evidence that social information, even when minimal, can guide leadership choice *based on* facial cues. These findings are the first to our knowledge to implicate a role of traits related to social and physical dominance in social transmission of face preferences, which has tended to focus on social transmission of attractiveness (e.g., Jones et al., 2007; Place et al., 2010; Little et al., 2015; see Gouda-Vossos et al., 2018 for a recent review). Our findings also extend prior work on sex differences in alliance formation based on facial cues (Watkins and Jones, 2016), revealing contextual specialization in how men and women respond to group leaders based on minimal information.

Of note, our data provide evidence that social information, in general, reduces appearance-driven biases toward leaders (i.e., our initial analysis of baseline changes across the sample). Within our model, social information reduces the salience of facial cues to high dominance and intelligence in out-group leaders, rather than generating substantive increases in preferences for these traits among in-group leaders (i.e., from baseline). In other words, as perceived dominance and intelligence becomes more or less important in evaluations of leaders and distinguishing individual leaders from one another, our findings raise the interesting possibility that people are evaluated differently as leaders depending on the media environment and/or the side they fall on in a socio-political debate, consistent with the “group-ish” mind-set (Haidt, 2012). However, this additional information (generally speaking) does not overwhelmingly *strengthen* biases toward candidates who are traditionally perceived as well-suited to a leadership role (Todorov et al., 2005; Ballew and Todorov, 2007; Little et al., 2007; Antonakis and Dalgas, 2009; Re et al., 2012, 2013; Olivola et al., 2014; Re and Perrett, 2014), at least when considering responses to standardized face photographs of potential leaders posing with neutral expressions.

Our priming manipulation may have limitations in priming the “new media environment” (e.g., Leetaru, 2011; Coviello et al., 2014; Kramer et al., 2014; see also Hill et al., 2010) as participants read lists of words rather than engage with genuine articles on important “hot button” issues. Our current design has advantages in terms of internal validity, however, as we are able to demonstrate that regardless of the socio-political issue our sample imagined, our “minimal manipulation” (Prentice and Miller, 1992; see also Baas et al., 2008 for discussion) had a direct effect on social judgments of leaders. Thus, effects of partisanship may be more substantial when engaging with this same issue in “higher-stakes” scenarios, or when reading an article where the writer’s partisanship is stronger (i.e., because the article is longer) but involves the same literary techniques, where two socio-political groups are portrayed as distinct on the valence and dominance dimensions and the reader’s prior beliefs are reinforced rather than challenged. Moreover, we control for the personal importance of the issue imagined. Genuine online articles, by contrast, may add noise if participants are influenced by the news outlet, the extent to which the issue has been resolved since publication, and effects of their political orientation on both prior knowledge and engagement with the issue they are reading about.

Other potential limitations are worthy of discussion. First, our lack of relationship between decisiveness and performance on the face judgment task may reflect a false negative finding in light of potentially inadequate power to detect such correlations if variation in decisiveness is low within the sample. Further work would likely prove fruitful, if the same phenomena were investigated in samples of children or cross-cultural populations, or were investigated using actual election data and candidate photographs, other implicit measures (e.g., Stillman et al., 2018), different contexts where sex-related biases in leadership perception may be more or less apparent (e.g., within certain work environments or “harsh” ecologies), or other “high-stakes” experimental scenarios. Indeed, given that we did not draw participants’ attention to the facial cues under examination and coded responses in order to measure the relative preference for high versus low facial cues to dominance and intelligence (before and after priming), it would be useful to examine whether these implicit associations between facial cues and leadership ability, and biases we hold toward people who “look like a leader”, change in a similar way when using well established tests of implicit association. As our brief priming manipulation (reading text varying in valence and dominance from a hypothetical online writer) and knowledge of the group membership of our pictured faces was sufficient to alter leadership judgments, effects in the real world could well be substantial or consequential, via repeated exposure to partisan sources (see, e.g., Hart et al., 2009; Haidt, 2012; Del Vicario et al., 2016) or misinformation (Lewandowsky et al., 2012) or when accompanied by other cues to intelligence or dominance such as vocal or behavioral cues. In light of the importance of small margins in swaying past election outcomes (see Bond et al., 2012 for discussion), our experimental results suggest that appearance and social information may afford strategic advantage for candidates or speakers in important debates, when two opponents present arguments of similar quality.

In sum, our research reveals that social knowledge of group membership reduces preferences for facial cues to high dominance and intelligence in out-group versus in-group leaders, with this bias greater when the media are *less* partisan. We also observed a general bias in the salience of women’s appearance, moderated by media partisanship, and a general in-group bias toward facial cues to high dominance among leaders of our own-sex (independent of media partisanship). Collectively, these first two findings suggest that contexts that are more likely to *challenge* versus re-inforce pre-existing beliefs (i.e., less partisan media) generate a relative bias toward in-group leaders based on facial characteristics typically associated with leadership, which could create a strategic advantage for our socio-political group. Our research highlights the sophisticated ways in which relatively minimal information shapes cognition in this context, which could have implications for leadership selection and social cohesion within groups at work, in the wider community or during national elections.

## Data Availability Statement

The datasets generated for this study can be found in the Open Science Framework (https://osf.io/utvnh/) with accompanying codebook.

## Ethics Statement

The research involved human participants was reviewed and approved by the University Teaching and Research Ethics Committee, University of St Andrews. Participants could only take part in this online study if they provided informed consent to participate.

## Author Contributions

CW wrote the manuscript and pre-registration report and analyzed the data. DX programmed the experiments. DP funded the experiments. DX and DP provided critical feedback on the manuscript and aspects of the experimental design and rationale.

## Conflict of Interest

The authors declare that the research was conducted in the absence of any commercial or financial relationships that could be construed as a potential conflict of interest.

The handling Editor declared a past collaboration, though no other collaboration with one of the authors, CW.

## Footnotes

1 https://osf.io/utvnh/
